# RNA N^6^-Methyladenosine Modifications and the Immune Response

**DOI:** 10.1155/2020/6327614

**Published:** 2020-01-21

**Authors:** Ya-Nan Wang, Chen-Yang Yu, Hong-Zhong Jin

**Affiliations:** Department of Dermatology, Peking Union Medical College Hospital, Chinese Academy Medical Science and Peking Union Medical College, Beijing, China

## Abstract

N^6^-methyladenosine (m^6^A) is the most important modification of messenger RNAs (mRNAs) and long noncoding RNAs (lncRNAs) in higher eukaryotes. Modulation of m^6^A modifications relies on methyltransferases and demethylases. The discovery of binding proteins confirms that the m^6^A modification has a wide range of biological effects and significance at the molecular, cellular, and physiological levels. In recent years, techniques for investigating m^6^A modifications of RNA have developed rapidly. This article reviews the biological significance of RNA m^6^A modifications in the innate immune response, adaptive immune response, and viral infection.

## 1. Background

Various chemical modifications of DNA or posttranslational modifications of proteins have been investigated for many years. However, studies of chemical modifications of RNA, which make up the “epitranscriptome,” are still in their infancy [1, 2]. Currently, the updated MODOMICS database includes 172 known RNA modifications. Among these modifications, N^6^-methyladenosine (m^6^A) is the most abundant nucleotide modification of messenger RNAs (mRNAs) and long noncoding RNAs (lncRNAs) in nearly all higher eukaryotes [3]. Here, we review the role of the m^6^A modification, especially in immune responses.

## 2. RNA m^6^A Modifications and Protein Factors

m^6^A, which refers to a modification occurring at the sixth position of adenine (A) bases in RNA, is widely found in yeast, plants, *Drosophila*, mammals, and viruses [4]. m^6^A modifications are highly conserved and are mainly confined to the following consensus sequence: RRACH (R = G or A; H = A, C, or U). m^6^A modifications are preferentially enriched in the long internal exons and 3′UTR regions of linear RNAs [5] and are reversible and involve various protein factors, including methyltransferases (“writers”), demethylases (“erasers”), and binding proteins (“readers”). First, writer enzymes mediate m^6^A mRNA modifications. METTL3 was the first writer identified as a component of a methyltransferase complex [6], and knockout of METTL3 in mouse embryonic stem cells (ESCs) significantly reduces the level of mRNA m^6^A modifications [5]. METTL14 is also a component of the methyltransferase complex. METTL3 and METTL14 bind tightly to each other; METTL3 contributes to the catalytic residue and METTL14 contributes to the structure of the catalytic centre and acts as an RNA-binding scaffold [7]. The METTL3/14 heterodimer interacts with Wilms' tumour 1-associated protein (WTAP) in the nucleus. WTAP is related to alternative splicing and localization of the heterodimer in nuclear speckles [7]. Hakai and virilizer (KIAA1429) appear to be WTAP-related components in mammals and can regulate m^6^A on RNA [8]. METTL16 is an independent human m^6^A methyltransferase that targets pre-mRNAs and various noncoding RNAs [9]. Second, enzymes called erasers mediate m^6^A demethylation on mRNA. The fat-mass and obesity-associated protein (FTO) was the first nuclear RNA m^6^A demethylase to be identified, and it preferentially demethylates the m_2_ isoform (N^6^,2′-O-dimethyladenosine, m^6^Am) rather than m^6^A. Moreover, FTO reduces the stability of m^6^Am mRNAs [10]. FTO is also associated with obesity, food intake, and energy metabolism [11]. ALKBH5 is another demethylase [12]; its knockdown in mice leads to an increase in m^6^A levels and impaired fertility arising from effects on spermatocyte apoptosis during meiotic metaphase [12]. Third, reader proteins specifically recognize m^6^A and participate in the degradation of downstream RNA as well as translation. Different readers produce different biological effects through different pathways. YTHDF1 and YTHDF3 recognize and bind to the modifications on RNA to directly modulate mRNA translation efficiency [13]. YTHDF2 mediates mRNA decay [14], while YTHDC1 modulates the affinity between splicing factors and RNA to influence RNA splicing [15]. YTHDC2 also enhances the translation efficiency of its targets and decreases their mRNA abundance [16]. Alarcon et al. found that the heterogenous nuclear ribonucleoprotein (hnRNP) A2/B1 is a “reader” of m^6^A that directly binds to m^6^A-modified RNAs [17]. However, Wu et al. reported that instead of directly binding to m^6^A-modified RNA, m^6^A promotes the accessibility of hnRNP A2/B1 to certain binding sites [18]. The discovery of methyltransferases and demethylases confirmed that the RNA m^6^A modification is dynamic and reversible, and the discovery of binding proteins confirmed that the m^6^A modification has a wide range of biological effects and significance [19]. Nonetheless, according to Ke et al., m^6^A modifications in newly formed pre-mRNAs is the same as those on nuclear and steady-state cytoplasmic mRNAs [20], which strongly opposes the proposed “dynamic” regulatory role of methylation and demethylation [20].

## 3. The Biological Functions of RNA m^6^A Modifications

The biological functions of RNA m^6^A modifications occur at three different levels: molecular, cellular, and physiological [21]. At the molecular level, an RNA modification posttranscriptionally regulates RNA splicing, transport, translation, stability, and localization [19]. YTHDC1 and HNRNP affect RNA splicing by interacting with other splicing factors [15]. mRNAs of the clock genes Per2 and Arntl exhibit slower export and a longer circadian period than the corresponding controls when the m^6^A modification is inhibited, indicating that the level of m^6^A in a target mRNA is critical for nuclear export and intracellular distribution [22]. YTHDF1 and YTHDF3 modulate translation efficiency by binding to m^6^A-modified target genes [13], and YTHDF2 affects mRNA degradation and stability [13]. At the cellular level, RNA m^6^A modifications determine the fate of mammalian embryonic stem cells (mESCs) [5]. Indeed, most transcripts of core pluripotency gene transcripts are targets of METTL3 [5, 23], and METTL3 knockout in mESCs and epidermal cells decreases the m^6^A level, promotes self-renewal, and inhibits cardiomyocyte and neuronal differentiation [5, 23]. Moreover, increasing the m^6^A abundance stimulates mouse embryonic fibroblast (MEF) reprogramming into pluripotent stem cells, whereas decreasing the m^6^A levels inhibits this reprogramming [24]. These findings suggest that the m^6^A modification plays a powerful and precise regulatory role in cell developmental programmes. At the physiological level, reversible m^6^A modification has various consequences. For example, overexpression of the demethylase FTO results in increased food intake and obesity [25]. In addition, studies have found that the m^6^A modification is related to neural stem cells, brain development, Parkinson's disease, and mental illness [26]. Furthermore, the m^6^A modification has been found to have an impact on tumour initiation and progression through various mechanisms, which have been covered in other reviews [27].

In contrast, there are few reports on m^6^A modifications of noncoding RNAs (ncRNAs), and the role of m^6^A modifications in ncRNA remains to be further investigated. However, it is known that lncRNA and miRNA function can be regulated by m^6^A. For example, He et al. proposed that ALKBH5 can inhibit the motility of pancreatic cancer by demethylating the lncRNA KCNK15-AS1 [28]; m^6^A-induced lncRNA RP11 can trigger the dissemination of colorectal cancer cells by posttranslationally upregulating Zeb1 [29]. In addition to the effects on lncRNAs, m^6^A modifications are associated with miRNA synthesis and function [17, 30]. For instance, METTL14 participates in adding m^6^A modifications to the pre-miRNA transcript of the antioncogene miR-126a [31]. Specifically, downregulation of METTL14 in hepatocellular carcinoma (HCC) is associated with reduced miR-126a levels and increased metastatic capacity [31]. Furthermore, the METTL3-miR-25-3p-PHLPP2-AKT pathway is associated with pancreatic transformation [32], and m^6^A modifications of AGO2 mRNA contribute to cellular ageing by regulating global miRNA synthesis [33]. It has also been reported that YTHDC1 recognizes m^6^A marks in mESCs and is essential for X-inactive specific transcript (XIST) activity [34]. Recently, it has also been found that enhancer RNAs (eRNAs), noncoding transcripts produced from enhancer regions that act as regulators of transcription, are highly m^6^A modified [35].

## 4. Techniques for Detecting m^6^A Modifications in RNA

In 1958, RNA was first shown to contain modifications, which play important roles in biological functions [36]. However, RNA m^6^A modifications do not change the nature of base pairing, and thus, m^6^A cannot be directly detected by sequencing. In fact, the lack of sensitive methods for detecting RNA m^6^A modifications has constrained scientific interest in this field. First, within the context of the rapid development of next-generation sequencing technologies, methylated RNA immunoprecipitation (Me-RIP) for identifying m^6^A sites on mammalian RNA emerged in 2012 [37]. Specifically, RNA samples are fragmented into 100-150 nucleotide (nt) segments, which are incubated with an anti-m^6^A polyclonal antibody; the mixture is immunoprecipitated by incubation with protein A beads, and the enriched m^6^A-containing pooled RNA and input RNA control are deep-sequenced [37]. Although this method is easy to perform, its resolution is approximately 200 nt, and the location of m^6^A sites cannot be identified at the single-nucleotide level. Second, single-molecule real-time RNA sequencing and site-specific cleavage and radioactive labelling followed by ligation-assisted extraction and thin-layer chromatography (SCARLET) can accurately determine the precise location of m^6^A at any site in mRNA/lncRNA with single-nucleotide resolution [38]. With this technique, m^6^A-containing candidate sites are specifically cleaved, and radiolabelling and site-specific ligation are then performed; the candidate residues containing m^6^A are separated from total RNA or polyadenylated mRNA (polyA+RNA) by nuclease digestion, and this mixture is subsequently analysed by thin-layer chromatography (TLC) [38]. The disadvantages of this technique are that it is time consuming and cannot identify the location of m^6^A sites on a global, transcriptome-wide level. Third, a newer single-nucleotide resolution method termed m^6^A individual nucleotide resolution crosslinking and immunoprecipitation (miCLIP) can accurately locate m^6^A loci in the whole transcriptome at a single-nucleotide resolution level without any pretreatment of cells containing modified nucleotides, such as 4-SU [39]. This technique crosslinks anti-m^6^A antibodies to RNA by ultraviolet (UV) radiation to form antibody-RNA crosslinks; reverse transcription of the crosslinked RNA results in highly specific mutations in the cDNA, which can be detected by sequencing and used to identify single m^6^A residues. However, the disadvantage of miCLIP is that it is not quantitative. Fourth, high-resolution melting (HRM), a high-resolution m^6^A mapping technique, directly detects base changes based on the melting characteristics of nucleic acids, allowing chemical or enzymatic information to be retained and avoiding sequence distortion [40]. Fifth, MAZTER-Seq employs the sequence-specific, methylation-sensitive bacterial single-stranded ribonuclease MazF to provide nucleotide resolution quantification of m^6^A methylation sites, though the method captures only 16% of the m^6^A (ACA-containing) sites in the mammalian system [41].

## 5. RNA m^6^A Modifications and the Innate Immune Response

The immune system is the most effective weapon against pathogen invasion. The immune system detects and removes foreign matter and microorganisms and maintains homeostasis. Immune dysfunction is involved in almost all known human diseases, including infection, inflammation, cancer, and metabolic syndromes. When a person's finger is injured by a large splinter containing many bacteria, within a few hours, the area near the entry point of the splinter becomes red and inflamed. These symptoms are indications that the innate immune system has been activated. In many instances, activation of the innate immune system is so effective and quick that the adaptive immune system is never activated. Mobilization of the adaptive system requires time because B and T cells must be specifically produced through the process of clonal selection and proliferation. Posttranscriptional RNA modifications regulate RNA splicing, transport, translation, stability, and localization, indicating that RNA modifications are likely to play an important role in the immune response. Indeed, studies have reported that RNA m^6^A modifications have biological significance for the innate immune response. First, m^6^A modifications have been demonstrated to promote dendritic cell (DC) activation and function [42], and depletion of METTL3 results in disruption of DC maturation [42]. Second, Toll-like receptors (TLRs) are critical proteins of the innate immune response that activate downstream effects by recognizing pathogen-associated molecules. It has been reported that m^6^A-modified RNA cannot activate TLR3, TLR7, or TLR8 [43, 44], and this inability may be related to impairment of the thermodynamic stability of RNA duplexes and lower immunogenicity [45]. Third, m^6^A modifications can suppress the antiviral innate immune system by reducing type I interferon production following pattern recognition receptor (PRR) binding to nonself nucleic acids, such as viral RNAs [46, 47]. Depletion of METTL14 inhibits virus reproduction by promoting both accumulation and stability of IFNB1 mRNA, although depletion of ALKBH5 reduces production of IFNB1 mRNA triggered by human cytomegalovirus (HCMV) or dsDNA, with no effect on DNA decay [46, 47]. In addition, m^6^A modifications have been demonstrated to further affect the expression of interferon-stimulated genes (ISGs) by repelling RNA-binding proteins such as G3BP1, G3BP2, and CAPRIN1 [48]. Fourth, m^6^A has been identified in the mRNA of genes encoding several essential molecules of the innate immune system, including TRAF3, TRAF6, and MAVS [49]. ALKBH5 recruited by the RNA helicase DDX46 removes m^6^A from the 3′UTRs of TRAF3, TRAF6, and MAVS mRNAs, resulting in decreased export of these transcripts from the nucleus [49].

## 6. RNA m^6^A Modifications and the Adaptive Immune Response

RNA m^6^A modifications are also strongly associated with T cells and the adaptive immune response. First, RNA m^6^A modifications determine cell fate transition in mESCs and help CD4^+^ T cells differentiate into various T helper (Th) subtypes under stimulation with cytokines and antigens [50]. Li et al. found that m^6^A regulates Th cell differentiation; in adaptive immune progenitor cells (naïve T cells), m^6^A targets the mRNA of the “gatekeeper” IL-7 signalling protein to control the homeostasis and differentiation of naïve T cells in response to various dynamic signals and external stimulation, ensuring homeostasis and expanding the biological effects of RNA m^6^A modifications [51]. Specifically, through the establishment of conditional knockout mouse models of METTL3 and METTL14, the IL-7-JAK1/STAT5 signalling pathway was found to be inhibited by inducing the degradation of mRNAs of the SOCS gene family, thus promoting both the differentiation of naïve T cells into Th1 and Th17 cells and T cell homeostatic proliferation [51]. These results suggest that m^6^A specifically controls the degradation rate of genes that respond immediately to various environmental stimuli to regulate T cell homeostasis and differentiation [51]. Second, cellular m^6^A modifications play a role in the generation and function of T regulatory cells (Tregs), which are an essential CD4^+^ subset of T effector cells that are responsible for immunosuppression in inflammation and in tumour microenvironments. A study by Tong et al. reported that conditional METTL3-knockout mice develop severe systemic autoimmune diseases, suggesting that the absence of m^6^A modifications induced loss of the suppressive function of Tregs [52]. Third, m^6^A modifications are involved in the crosspresentation of tumour antigens [53]. YTHDF1 promotes the translation of lysosomal cathepsins by binding to transcripts encoding lysosomal proteases marked by m^6^A [53]. Therefore, the absence of YTHDF1 results in enhanced crosspresentation of tumour antigens [53]; YTHDF1 also promotes crosspriming between CD8^+^ T cells and antigen-presenting cells (APCs), and this process relies mainly on DCs [53]. In addition, a higher level of CD8^+^ cell infiltration was observed in biopsies of patients with reduced YTHDF1 expression [53]. Fourth, it has been reported that FTO is involved in the dopamine signalling process [54]. Dopamine receptors (D1- and D2-like receptors) are expressed not only in the brain but also in T cells and have key roles in mediating T cell function and the development of thymic T cells [55, 56], suggesting that FTO may affect the function of T cells through dopamine receptors. Casalegno-Garduno et al. found that WTAP elicits serological and cellular immune responses in patients with leukaemia, which further demonstrates the correlation between RNA m^6^A modifications and T cells [57]. Because T cells participate in the entire adaptive immune response, the effects of m^6^A modifications on T cells have broad implications for the adaptive immune response and may be involved in the development and progression of various immune-related diseases. The m^6^A eraser ALKBH5 is highly expressed in immune-rich organs such as the spleen and lungs [12]. Moreover, IL-6 and IL-8 can be induced in keratinocytes by dopamine receptors [58]. These findings further emphasize the relevance of RNA m^6^A modifications in immune responses.

## 7. RNA m^6^A Modifications and Viral Infection

Recent works have demonstrated that m^6^A modifications are involved not only in the life cycle of the virus but also in the host response to viral infection, playing either a proviral or an antiviral role. First, m^6^A modifications in the Rev response element (RRE) of HIV promote binding between Rev and viral RNA, leading to enhanced viral RNA export [59]. Consistently, YTHDF binding proteins are upregulated in HIV-1 infection [60]. YTHDF is also a negative regulator because it suppresses viral proliferation by binding to viral genomic RNA and reducing HIV reverse transcription products, indicating that the function of m^6^A modifications depends on different stages of the viral life cycle [61]. Second, a proviral role has also been observed with influenza A virus. Although the mechanism remains uncertain, one assumption is that YTHDF2 promotes the degradation of antiviral gene transcripts [62]. Third, m^6^A modifications play a positive role in enterovirus 71 (EV71) replication [63]. Fourth, m^6^A modifications impair viral replication by blocking the packaging of viral RNA into new virions during flavivirus infections [64]. Flaviviruses, such as the Zika virus (ZIKV), hepatitis C virus (HCV), and dengue virus, are positive-sense single-stranded viruses. Fifth, DNA viruses, including Kaposi sarcoma-associated herpesvirus (KSHV), have also been studied, although the function of m^6^A modifications in KSHV remains controversial. One study reported that the binding protein YTHDF2 impairs KSHV lytic replication by promoting the degradation of viral gene transcripts [65], and another study elucidated a proviral role for m^6^A modifications, reporting that YTHDC1 is able to facilitate KSHV lytic replication by inducing the splicing of the replication transcription activator (RTA) [66]. Further research in this field may help to develop new antiviral therapies.

## 8. Conclusion

Currently, 172 different modifications of RNA molecules have been identified. However, the lack of a sensitive technique for the detection of RNA modifications has resulted in limited scientific research in this area. The modulation of m^6^A modifications relies on methyltransferases and demethylases, and the discovery of binding proteins has confirmed that m^6^A modifications have a wide range of biological effects and significance. By summarizing the existing literature, we found that m^6^A modifications are widely involved in the immune response, including innate immune responses, adaptive immune responses, and viral infection. Compared to studies in tumour research, studies on RNA m^6^A modifications in immunity are lacking. With the rapid development of technologies to detect RNA m^6^A modifications, future studies will help to reveal the correlation between RNA m^6^A modifications and immune diseases.
